# Peroral endoscopic myotomy compared to laparoscopic Heller myotomy and pneumatic dilation in the treatment of achalasia: a systematic review

**DOI:** 10.1093/dote/doad055

**Published:** 2023-08-03

**Authors:** Adam North, Nilanjana Tewari

**Affiliations:** Warwick Medical School, University of Warwick, Coventry, UK; General Surgery Department, University Hospitals of Derby and Burton, Derby, UK

**Keywords:** achalasia, benign esophageal diseases, dilation, diseases of the esophagus, endoscope, endoscopic mucosal resection, endoscopic submucosal dissection, endoscopic surgery, treatment

## Abstract

Peroral endoscopic myotomy (POEM) is an intervention for the treatment of achalasia which has gained popularity over the last decade. It’s efficacy and invasiveness are comparable to laparoscopic Heller myotomy (LHM). The purpose of this systematic review is to compare POEM to existing therapies. The systematic review was performed following the preferred reporting items for systematic reviews and meta-analyses (PRISMA) guidelines. MEDLINE, EMBASE, Web of Science and Cochrane Libraries were searched using keywords: esophageal achalasia, POEM, LHM, pneumatic dilation (PD), and related terms. The studied outcomes were Eckardt score, clinical success, hospital stay, cost-utility analysis, complications, and post-treatment gastro-esophageal reflux disease. Articles were reviewed by one researcher and uncertainty was resolved by a second researcher. The search strategy retrieved 1948 citations. After removing duplicates and applying the exclusion criteria, 91 studies were selected for full-text review of which a total of 31 studies were considered eligible for further analysis, including two studies which were found through manual searching. POEM has improved efficacy compared to PD with similar cost-effectiveness. POEM results showed comparable patient outcomes when compared with laparoscopic myotomy. Overall, POEM is a feasible first-line treatment for achalasia.

## BACKGROUND

Achalasia is an esophageal motility disorder characterized by a failure of relaxation of the lower esophageal sphincter.^1^ Its presentation is characterized by progressive dysphagia to solids and liquids. Additional symptoms include retrosternal chest pain, regurgitation, and weight loss.^2^

Current pharmacological therapies have little effectiveness beyond the short term and their use is limited by significant side effects.^3^ Endoscopic therapies are available. Botulinum toxin can be injected into the lower esophageal sphincter. This provides better efficacy than pharmacological treatments but needs to be repeated at intervals as the effects wane.^3^ Pneumatic dilation (PD) involves using an endoscopic balloon to disrupt the sphincter muscle fibers with a circular force. This treatment provides good symptom relief but usually leads to recurrence.^4^ The disadvantage of endoscopic treatments is the need for repeated treatment.

The gold standard treatment for achalasia is laparoscopic Heller myotomy (LHM). This surgical technique involves exterior myotomy of the lower esophageal sphincter and is usually performed with an anti-reflux procedure.^4^ It is as effective as pneumatic dilatation and longer lasting.^5^^,^^6^ Peroral endoscopic myotomy (POEM) was first described in humans in 2009 and involves dissection of the lower esophageal mucosa via an endoscopic approach.^7^ It is thought that POEM may offer the long-term efficacy of Heller myotomy.^8^ This literature review aims to compare medium to long-term efficacy of POEM.

## METHODS

A systematic review of the literature was undertaken following the PRISMA guidelines. MEDLINE, EMBASE, Web of Science and Cochrane Library databases were searched for POEM studies using the keywords: esophageal achalasia, POEM, LHM, PD, and related terms. Articles published from January 2008 to December 2021 in English were included using search filters. Searches were undertaken on 11 November 2021 and follow up search on 22 July 2022. Manual searching was undertaken of Pubmed database and bibliographies of included studies. Inclusion and exclusion criteria are detailed in Table 1. Papers were reviewed by one researcher and any uncertainty was resolved by a second researcher. Analysis included: (1) baseline characteristics of studies: first author, year of publication, study duration, study design, number of patients and interventions compared and (2) clinical outcomes of studies: pre- and post- Eckardt score, clinical success, hospital stay, cost-utility analysis, complications, and post-treatment GORD. Eckardt score is detailed in Table 2.

**Table 1:** Inclusion and exclusion criteria

Inclusion criteria	Exclusion criteria
English language	Systematic review or meta-analysis
Comparison of POEM and either LHM or PD	Pediatric studies
Adequate follow up period of at least 3 months	Robot assisted myotomy

LHM, laparoscopic Heller myotomy; PD, pneumatic dilation; POEM, peroral endoscopic myotomy.

**Table 2:** Eckardt scoring system for achalasia^3^

Score	Symptom			
	Weight loss (kg)	Dysphagia	Retrosternal pain	Regurgitation
0	None	None	None	None
1	<5	Occasional	Occasional	Occasional
2	5–10	Daily	Daily	Daily
3	>10	Each meal	Each meal	Each meal

Medium-long term efficacy was defined as efficacy at greater than 12-month follow-up. Short term follow-up was defined as results obtained within the 12-months following intervention.

Critical appraisal of papers was performed to assess risk of bias using CASP tools.

## RESULTS

**Fig. 1:**
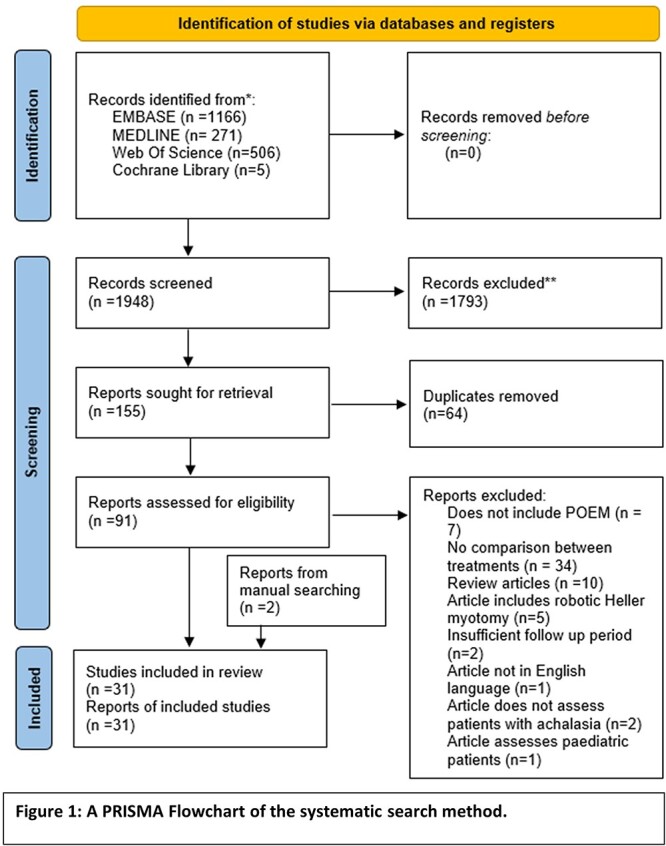
A PRISMA Flowchart of the systematic search method.

A PRISMA flowchart of this systematic review is included in Fig. 1. Following application of inclusion and exclusion criteria and manual searching, 31 studies were included in review.

**Fig. 2:**
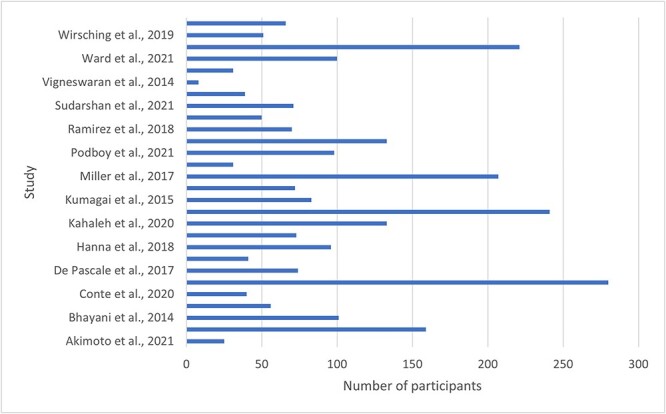
Total number of participants included in each study. Khoraki et al., 2021 and Trieu et al., 2021 were excluded from this figure due to the large scale nature of database searching.

Details of each article included in the analysis are shown in Table 3. Following critical appraisal, no articles exhibited high risk of bias. Fig. 2 details the number of participants investigated in each individual study.

**Table 3:** Summary of details of articles included in systematic review

**Study**	**Year of Publication**	**Country**	**Intervention**	**Duration of study (months)**	**Study design**	**Number of participants (*n*)**
Akimoto *et al*.	2021	Japan	POEM vs. LHM	278	Retrospective	25
Attaar *et al*.	2021	USA	POEM vs. LHM	116	Retrospective	159
Bhayani *et al*.	2014	USA	POEM vs. LHM	72	Prospective	101
Chan *et al*.	2016	Hong Kong	POEM vs. LHM	180	Retrospective	56
Conte *et al*.	2020	Brazil	POEM vs. LHM	—	RCT	40
Costantini *et al*.	2020	Italy	POEM vs. LHM	48	Retrospective	280
De Pascale *et al*.	2017	Italy	POEM vs. LHM	40	Retrospective	74
Greenleaf *et al*.	2018	USA	POEM vs. LHM	6	Retrospective	41
Hanna *et al*.	2018	USA	POEM vs. LHM	60	Retrospective	96
Hungness *et al*.	2013	USA	POEM vs. LHM	99	Prospective vs. Retrospective	73
Kahaleh *et al*.	2020	International	POEM vs. LHM	60	Retrospective	133
Khoraki *et al*.	2021	USA	POEM vs. LHM	39	Public Database Searching	11,270
Kim *et al*.	2019	Korea	POEM vs. PD	331	Retrospective	241
Kumagai *et al*.	2015	Sweden	POEM vs. LHM	19	Prospective vs. Retrospective	83
Meng *et al*.	2017	China	POEM vs. PD	44	Retrospective	72
Miller *et al*.	2017	USA	POEM vs. LHM vs. PD vs. BI	60	Retrospective	207
Peng *et al*.	2017	China	POEM vs. LHM	48	Retrospective	31
Podboy *et al*.	2021	USA	POEM vs. LHM	48	Retrospective	98
Ponds *et al*.	2019	International	POEM vs. PD	40	RCT	133
Ramirez *et al*.	2018	Argentina	POEM vs. LHM	69	Prospective vs. Retrospective	70
Schneider *et al*.	2016	Sweden	POEM vs. LHM	49	Retrospective	50
Sudarshan *et al*.	2021	USA	POEM vs. LHM	64	Retrospective	71
Trieu *et al*.	2021	USA	POEM vs. LHM	12	Public Database Searching	3430
Ujiki *et al*.	2013	USA	POEM vs. LHM	46	Prospective	39
Vigneswaran *et al*.	2014	USA	POEM vs. LHM	33	Prospective	8
Wang *et al*.	2016	China	POEM vs. PD	72	Retrospective	31
Ward *et al*.	2021	USA	POEM vs. LHM	60	Retrospective	100
Werner *et al*.	2019	International	POEM vs. LHM	35	RCT	221
Wirsching *et al*.	2019	USA	POEM vs. LHM	48	Prospective	51
Zheng *et al*.	2019	China	POEM vs. PD	43	Retrospective	66

LHM, laparoscopic Heller myotomy; PD, pneumatic dilation; POEM, peroral endoscopic myotomy; RCT, randomized controlled trial.

### Medium-long term efficacy

Efficacy is improved following POEM compared to pneumatic dilation,^9–12^ as evidenced by observation of statistically significant symptom improvements in a 133-patient randomized controlled trial (RCT)^9^ at 2 years and in 2 retrospective studies.^10^^,^^11^ In addition, statistically significant improvements in treatment success rates (100 vs. 50% with Eckardt <3) were noted in type III achalasia patients retrospectively at 1 year follow-up^12^.

POEM is of similar efficacy to LHM in follow-up.^13–19^ Comparable treatment success was observed by two RCTs^13^^,^^14^ on analysis of 221 and 40 patients. A retrospective analysis of 98 patients observed significantly longer time to treatment failure in POEM groups compared to LHM despite no difference in Eckardt scores at 36 months.^20^ Additionally, significantly improved integrated resting pressures were observed in POEM patients without reciprocal improvements in Eckardt scores following retrospective analysis of 71 patients. ^21^ A multicentre retrospective analysis of 133 patients noted significantly improved Eckardt and lower esophageal sphincter pressures in POEM patients.^22^

### Short term efficacy

Efficacy is improved following POEM compared to pneumatic dilation^10,11,12,23,^. Significantly lower Eckardt scores were observed in POEM patients compared with patients undergoing PD in retrospective studies.^10^^,^^12^ Other retrospective studies observed statistically similar treatment success rates.^11^^,^^23^

Treatment success is comparable between POEM and LHM.^14^^,^^24–33^ A RCT noted no significant difference in Eckardt scores or basal LOS pressure at 6-month follow-up^14^. A number of prospective unrandomized studies observed comparable Eckardt scores at follow-up.^24–26^ These findings were reflected in retrospective analyses.^27–33^ Comparable barium esophagogram measurements of column height and percentage esophageal emptying were observed in another study.^29^

### Complications and re-treatment

Complication rates are comparable following POEM and PD.^11^^,^^23^ In the studies that assessed this, cumulatively there was one adverse event in the POEM groups compared to zero in the PD groups and there was no statistically significant difference in complication rates.

Complication rates are higher following POEM compared with LHM.^13^^,^^16–20^^,^^22^^,^^24^^,^^29–31^^,^^33^^,^^34^^,^^36^ A database search of over 3000 admissions in the USA noted no difference in adverse events.^34^ However, a similar study of over 11,000 patients found significantly higher rates of adverse events following POEM compared with LHM^35^. An RCT from Brazil observed statistically higher complication rates following POEM compared with LHM (64.3 vs. 35.7%).^14^ A larger RCT noted no statistically significant difference in complication rates following intervention with POEM compared with LHM^13^. A number of non-randomized studies concluded that complication rates in POEM and LHM were comparable.^16–20^^,^^22^^,^^24^^,^^29–31^^,^^33^^,^^36^

PD has higher rates of symptom recurrence than POEM, leading to higher rates of re-treatment.^9^^,^^10^^,^^12^ Significantly higher rates of re-treatment were noted in a multicentre RCT (PD = 46%, POEM = 8%).^9^ This was mirrored in a retrospective study from Korea (PD = 45.2%, POEM = 7.8%).^10^ Another retrospective study noted significantly more PD patients experienced symptom recurrence compared to POEM patients at 36 months (40% vs. 7%).^12^

LHM and POEM lead to comparable symptom recurrence and re-treatment rates.^18^^,^^20^^,^^28^^,^^33^ Significantly higher rates of re-intervention were noted in LHM patients compared to POEM patients in a multicentre retrospective analysis (40% vs. 14%).^22^ A number of retrospective studies noted no statistically significant difference in treatment failure rates or re-treatment.^18^^,^^20^^,^^28^^,^^33^

### Gastro-oesophageal reflux disease

POEM may lead to higher rates of gastro-esophageal reflux disease (GORD) symptoms than PD.^9^^,^^11^ A multicentre RCT observed significantly higher rates of GORD symptoms in POEM patients at 24-month follow-up (41 vs. 7%).^9^ A retrospective analysis observed comparable ‘classical’ GORD symptoms but significantly higher rates of mild heartburn in POEM patients 12 months after treatment.^11^ Another retrospective analysis noted comparable rates of GORD symptoms greater than 24 months post-treatment.^12^

POEM leads to higher rates of GORD symptoms after treatment compared with LHM.^13^^,^^14^^,^^16^^,^^19^^,^^30^ Significantly higher rates of GORD symptoms were noted in POEM patients combined with significantly more cases of endoscopic esophagitis at 24-month follow-up in a large RCT (Odds ratio 2.00).^13^ Another RCT observed significantly higher rates of esophagitis symptoms and endoscopy findings in POEM patients.^14^ A retrospective analysis noted significantly higher rates of esophagitis on endoscopy that was not reflected in reported GORD symptoms.^19^ A small retrospective analysis of patients who had previously undergone a LHM with fundoplication observed higher rates of GORD symptoms in POEM patients despite an existing anti-reflux procedure.^30^ Despite this, a number of observational analyses found comparable rates of GORD symptoms between the groups.^15^^,^^16^^,^^18^^,^^20^^,^^24^^,^^26^^,^^28^^,^^29^^,^^32^^,^^33^

### Cost-effectiveness

POEM leads to significantly higher treatment-associated costs than PD, but the cost-effectiveness of PD diminishes over time.^23^^,^^37^ A retrospective financial analysis in the USA concluded that PD was significantly more cost-effective than POEM initially, but that cost-effectiveness became comparable after 4 years due to retreatment.^37^ A retrospective analysis of 31 patients observed significantly higher hospitalization costs per patient for POEM compared to PD ($2620.3 vs.$1212.6, *P* value <0.001).^23^

POEM is similar to LHM in terms of cost-effectiveness.^17^^,^^24^^,^^35^^,^^37^^,^^38^ An American retrospective analysis concluded that POEM incurred significantly fewer total charges compared to LHM^30^. However, database searching of over 3000 admissions found significantly higher charges assigned to POEM patients than LHM patients.^34^ A similar study of over 11,000 patients noted comparable associated costs.^35^ This finding was reflected in a number of other studies.^17^^,^^24^^,^^37^^,^^38^ Conte *et al*.^14^ reported a small RCT which found significantly higher cost-per-patient in the POEM group compared to the LHM group.

### Length of hospital stay

Length of stay is statistically significantly shorter following PD than POEM.^12^ A retrospective analysis of 72 patients in China noted significantly shorter length of stay in PD patients post procedure (3 days vs. 8 days).

POEM results in shorter length of hospital stay compared to LHM.^14^^,^^16^^,^^20^^,^^26–28^^,^^38^ A randomized controlled trial found significantly shorter length of stay in the POEM cohort (3.7 days vs. 5.4 days, *P* value = 0.009).^14^ Multiple retrospective studies found that significantly shorter hospital stay was seen after POEM compared with LHM (2 days vs. 3 days, *P* value <0.001),^16^ (1 day vs. 2 days),^27^ (2 days vs. 3 days, *P* value = 0.0014).^19^ Similar findings of shorter hospital stay in POEM were noted in a number of other observational studies.^20^^,^^22^^,^^26^^,^^28^^,^^38^ Other observational studies have reported no difference in stay.^25^^,^^29^^,^^31^^,^^33^^,^^35^^,^^36^

## DISCUSSION

POEM results in a marked improvement in achalasia symptoms following treatment with benefit for an extended length of time.^9–12^^,^^23^ Effective symptom improvement has been evidenced in studies with large cohorts.^9^^,^^10^ This improvement appears to be especially beneficial in type III achalasia patients, the subtype that poses significant difficulties in treatment.^10^^,^^12^^,^^39^ Additionally, this improvement in treatment success is not accompanied by an increased likelihood of adverse events. This suggests POEM is a safe and effective treatment compared to pneumatic dilation.

Furthermore, POEM appears to be more likely to result in long lasting benefit without the need to undergo additional intervention.^9^^,^^10^^,^^12^ Significantly, almost half of PD patients experienced symptom recurrence during follow-up that required retreatment, compared to fewer than 10% of POEM patients. However, it appears that POEM patients go on to experience post-operative symptoms of gastro-esophageal reflux.^9^ The findings in this study may be accounted for by the use of a single session of pneumatic dilatation compared to the accepted practice of graded dilatations.^9^ It is understood that treatment related costs are higher in POEM.^40^^,^^41^ However, dedicated financial analysis concluded that cost-effectiveness of PD reduces to comparable levels due to the requirement for serial dilations.^37^ It can be deduced that POEM is a superior choice than PD due to the superior efficacy, particularly in the long term, and relative cost-effectiveness. Once initial costs of equipment sourcing are covered, superior patient outcomes can be obtained for costs similar to pneumatic dilation.^37^

Treatment of achalasia with POEM results in equivalent patient outcomes when compared to LHM. Patient outcomes at all timepoints appear to be comparable.^13–19^^,^^24–33^ This finding includes patients with type III achalasia where comparable patient outcomes were noted.^13^^,^^15^ It appears that the safety of POEM is not inferior to that of LHM.^34^^,^^35^ The finding of a significantly higher rate of recurrent symptoms in LHM patients compared to POEM in a multicentre analysis is contrasted by converse findings in a randomized controlled trial.^14^^,^^22^

POEM patients do experience GORD symptoms. This finding was highlighted by significantly higher rates of patient reported symptoms and endoscopic findings of esophagitis.^13^^,^^14^ Despite the presence of an existing anti-reflux procedure, POEM still leads to esophageal reflux and associated symptoms.^30^^,^^42^ These symptoms may be manageable conservatively compared to symptoms of achalasia through the use of proton pump inhibitors.^43^^,^^44^ POEM does not demand significantly higher funding than LHM and results in comparable cost-effectiveness.^35^^,^^37^ These findings suggest that POEM is non-inferior to LHM in terms of symptom relief and recurrence, and the increased development of GORD can be controlled medically.

It should be noted that POEM and LHM require significant skill and experience to be carried out effectively.^45^^,^^46^ Studies suggest comparable learning curves for these techniques. Due to the relatively uncommon nature of these interventions, many institutions may not see benefit in adoption of these as first line treatments due to the timeframe of training.

A number of limitations are present in this study. Most study designs included are retrospective without matching, introducing the possibility of bias. This may be improved in future literature as POEM is adopted as a treatment standard and randomization of treatment is justified. Few of the included studies undertook follow-up of POEM patients beyond 24 months, this was often compared to longer follow-up in LHM and PD patients leading to potential missed recurrence in POEM patients. Financial implications were considered in this study, however, no formal economic model was formulated.

Future recommendations include further RCTs to compare the outcomes of POEM compared to LHM/PD with reduced chance of bias. Additionally, long term follow-up of current studies would be beneficial to investigate the extended long-term outcomes of POEM. Further, review of the literature in pediatric and teenage cases may be beneficial as this age group would be most impacted by long-term post-operative esophageal reflux.

## CONCLUSIONS

This review has compared the clinical outcomes of achalasia patients following POEM procedure compared to PD or LHM patients. POEM has improved efficacy compared to PD with similar cost-effectiveness. POEM results in comparable patient outcomes compared to LHM. POEM patients do experience an increased rate of post-operative GORD. However, this can be managed effectively medically long-term. Further RCTs into this topic may result in confirmation of these findings and lead to adoption of POEM as a standard treatment for achalasia in patients that can tolerate general anesthetic.

## Author contributions

Nila Tewari (Writing—review & editing).

## Notes

Financial support: None
